# Soyasaponin β-glucosidase confers soybean resistance to pod borer (*Leguminivora glycinivorella*)

**DOI:** 10.1007/s42994-025-00214-7

**Published:** 2025-05-10

**Authors:** Chengyong Feng, Xindan Xu, Jia Yuan, Mingyu Yang, Fanli Meng, Guodong Wang

**Affiliations:** 1 State Key Laboratory of Seed Innovation, Institute of Genetics and Developmental Biology, Chinese Academy of Sciences, Beijing, 100101 China; 2 State Key Laboratory of Black Soils Conservation and Utilization, Key Laboratory of Soybean Molecular Design Breeding, Northeast Institute of Geography and Agroecology, Chinese Academy of Sciences, Harbin, 150081 China; 3 College of Advanced Agricultural Sciences, University of Chinese Academy of Sciences, Beijing, 100039 China; 4 Kunming Institute of Botany, Chinese Academy of Sciences, Kunming, 650201 China

**Keywords:** Soybean, Soyasaponin, β-glucosidase, Soybean pod borer, Homeostasis

## Abstract

Plant specialized metabolites are commonly stored in glycosylated forms within plant cells, with their homeostasis regulated by glycosyltransferases and β-glucosidases (BGLUs, also known as β-glucoside hydrolases (E.C.3.2.1.21)). Soyasaponins, the predominant triterpenoid compounds (C30) in soybean seeds, contain two sugar moieties attached at the C3 and C22 positions. While glycosyltransferases involved in soyasaponin biosynthesis have been well characterized, the role of BGLUs in soyasaponin homeostasis remains unclear. In this study, we identified *GmSSBG1* (*Soyasaponin β-glucosidase1*; *Glyma.07G258700*) as a candidate gene potentially involved in soyasaponin homeostasis through gene to gene co-expression analysis. Biochemical assays demonstrated that GmSSBG1 specifically hydrolyzes arabinose residues at the C22 position of A0- and B0-series soyasaponins. Loss-of-function mutations in *GmSSBG1* led to a significant accumulation of A0- and B0-series soyasaponins in mutant seeds, which correlated with a pronounced decrease in resistance to the soybean pod borer (*Leguminivora glycinivorella*). Our findings provide critical insights into the regulatory mechanisms underlying soyasaponin homeostasis and lay a theoretical foundation for molecular breeding strategies aimed at developing soybean lines with enhanced resistance to soybean pod borer, even to other insect pests.

**Supplementary Information:**

The online version contains supplementary material available at 10.1007/s42994-025-00214-7.

## Introduction

Triterpenes are among the most numerous and diverse groups of plant natural products (Thimmappa et al. 2014), with many well-known triterpenoid specialized metabolites occurring in glycosylated forms, like ginsenosides in ginseng (*Panax ginseng* (Shin et al. 2015)), glycyrrhizin in licorice (Jiang et al. 2020), and soyasaponins in soybean (Yuan et al. 2023). Glycosylation, a common chemical modification, involves the attachment of one or more sugar moieties to the triterpene skeleton. This process not only contributes to the chemical diversity of triterpenoids, but also plays a critical role in regulating their functional properties and ecological relevance. Glycosylation improves the chemical stability and water solubility of triterpenes, facilitates their storage and transport within plant cells, and underscores their importance in plant metabolism and adaptation (Louveau and Osbourn 2019). In plants, the glycosylation of specialized metabolites is often reversible due to the presence of the large number of glycoside hydrolases (Minic 2008). This reversibility is particularly relevant in *O*-glycosylation reactions. It is widely recognized that glycosylation reduces the toxicity of triterpenoid aglycones to plant cells themselves, allowing for their accumulation in significant quantities. Conversely, glycoside hydrolases mediate the release of the sugar moiety, generating more physiologically active triterpene aglycones, which play crucial roles in responding to pathogens, pests, and other (a)biotic stresses (Lacchini et al. 2023).

Among glycoside hydrolases, β-glucosidases (BGLUs) represent one of the largest families in plant. They play a key role in maintaining the balance between bioactive and storage form of specialized metabolites, including benzoxazinoids, glucosinolates cardenolides, and saponins. Under normal growth condition, BGLU enzymes and their glucosylated substrates are spatially separated within different subcellular compartments or distinct cells. However, upon herbivore attack or pathogen infection, the physical barriers between BGLUs and their glycosylated substrates are disrupted, leading to their rapid interaction. These results in the release of bioactive aglycones, which activate chemical defenses (Morant et al. 2008; Stathaki et al. 2024). This mechanism is commonly referred to as a two-component chemical defense system (Morant et al. 2008).

**Fig. 1:**
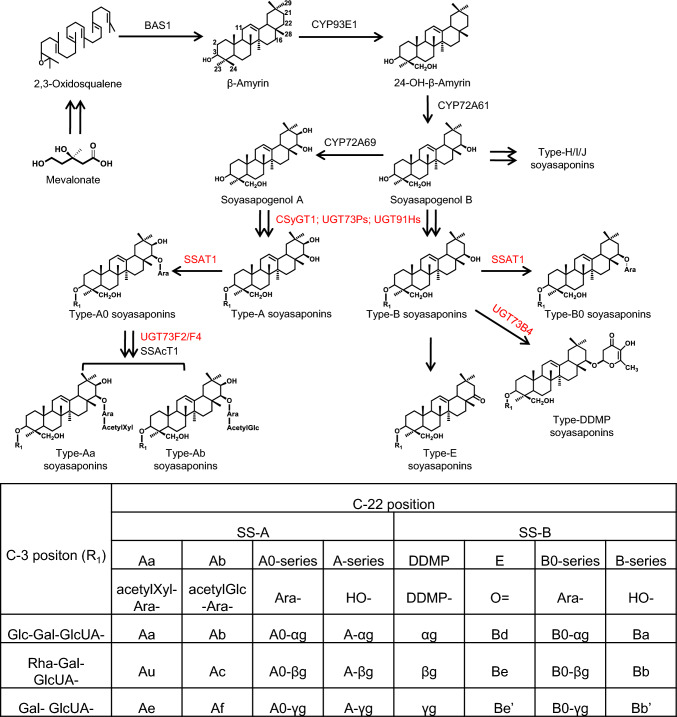
Proposed biosynthetic pathways of soyasaponins catalyzed by characterized enzymes in *Glycine max* L. The enzymes catalyzes the glycosylation reactions were highlighted in red. Double arrows represent multiple reactions. BAS1, β-amyrin synthase; CSyGT1, cellulose-synthase superfamily-derived glycosyltransferase1; SSAT1, soyasaponin arabinosyltransferase1; SSAcT1, soyasaponin acetyltransferase1

Soyasaponins, a class of oleanane-type pentacyclic triterpenes, can be primarily categorized into type-A, type-B, and type-E saponins based on the hydroxylation patterns of their aglycone backbones, which are glycosylated derivatives of soyasapogenol A, soyasapogenol B, and soyasapogenol E, respectively (Fig. 1) (Kudou et al. 1992). Similar to other triterpene biosynthesis pathways in plants, the biosynthesis of soyasaponins initiates from the mevalonic acid (MVA) pathway in the cytoplasm (Thimmappa et al. 2014). The biosynthesis of soyasaponins involves two major steps. The first step is the synthesis of sapogenins. Oxidosqualene is cyclized to β-amyrin by β-amyrin synthase1 in soybean. Hydroxylation of β-amyrin at specific positions generates various soyasapogenols, which includes hydroxylation at the C-24 position by CYP93E1 (Shibuya et al. 2006), at the C-22 position by CYP72A61 (Fukushima et al. 2013), and at the C-21 position by CYP72A69 (Yano et al. 2017). The second step involves glycosylation of soyasapogenols to form soyasaponins. Glycosyltransferases involved in soyasaponin biosynthesis in soybean have been well-investigated. The sequential glycosylation at the C-3 position involves the addition of glucuronic acid (1st sugar), galactose or arabinose (2nd sugar), and rhamnose or glucose (3rd sugar), which catalyzed by GmCSyGT1 (Chung et al. 2020), UGT73P2 or UGT73P10, and UGT91H4 or UGT73H9, respectively (Shibuya et al. 2010; Takagi et al. 2018; Yano et al. 2018). In group A soyasaponins, the first sugar attached at the C-22 position is arabinose, catalyzed by SSAT1 (Louveau et al. 2018). UGT73F2 or UGT73F4 further add glucose or xylose, respectively, to the arabinose moiety (Sayama et al. 2012). Regarding DDMP (2,3-dihydro-2,5-dihydroxy-6-methyl-4H-pyran-4-one) soyasaponins, the DDMP group was attached to C-22 position by a glycosyltransferase UGT73B4 (Sundaramoorthy et al. 2019). Recently, the soyasaponin acetyltransferase GmSSAcT1, responsible for the full acetylation of terminal sugars at the C-22 position in group A soyasaponins, has been functionally characterized (Yuan et al. 2023). Additionally, the vacuolar transporter GmMATE100, which mediates the transport of glycosylated soyasaponins into vacuoles, has been identified. Notably, GmMATE100 does not transport non-glycosylated soyasapogenols (Ma et al. 2024). Compared to the well-studied glycosyltransferases, the involvement of glucosidases in soyasaponin production in soybeans remains largely unexplored.

The soybean pod borer (SPB, *Leguminivora glycinivorella*), is one of the most significant pests affecting soybean production. The larvae of this pest feed exclusively on developing seeds within soybean pods, typically invading them during the flowering to maturation stages of soybean plants (Turnipseed and Kogan 1976; Chen et al. 2022). This feeding behavior disrupts seed development or causes premature seed death, leading to substantial reductions in soybean yield and quality (Edmonds et al. 2000). The damage caused by the SPB is particularly severe in major soybean-producing regions such as Northeast China, Japan, and North Korea, where it poses a serious threat to agricultural production and economic development (Zhao et al. 2015). Although it is commonly assumed that soybean specialized metabolites, including the abovementioned soyasaponins and the well-known isoflavonoids, may play a role in resistance to SPB infestation (Nozzolillo et al. 1997; Zhao et al. 2015), there is currently a lack of experimental evidence to support this hypothesis.

In this study, we identified a BGLU enzyme from soybean, named GmSSBG1 (Soyasaponin β-glucosidase 1; encoded by *Glyma.07G258700*). This enzyme specifically hydrolyzes arabinose residue at the C22 position of A0- and B0-series soyasaponins, while showing no activity toward soyasaponins with two sugars attached at the C22 position. Furthermore, knockout of *GmSSBG1* increased the susceptibility of soybean plants to damage caused by soybean pod borer (SPB), which provides an alternative strategy for sustainable pest control.

## Results

### GmSSBG candidate genes determined by gene to gene co-expression analysis

**Fig. 2:**
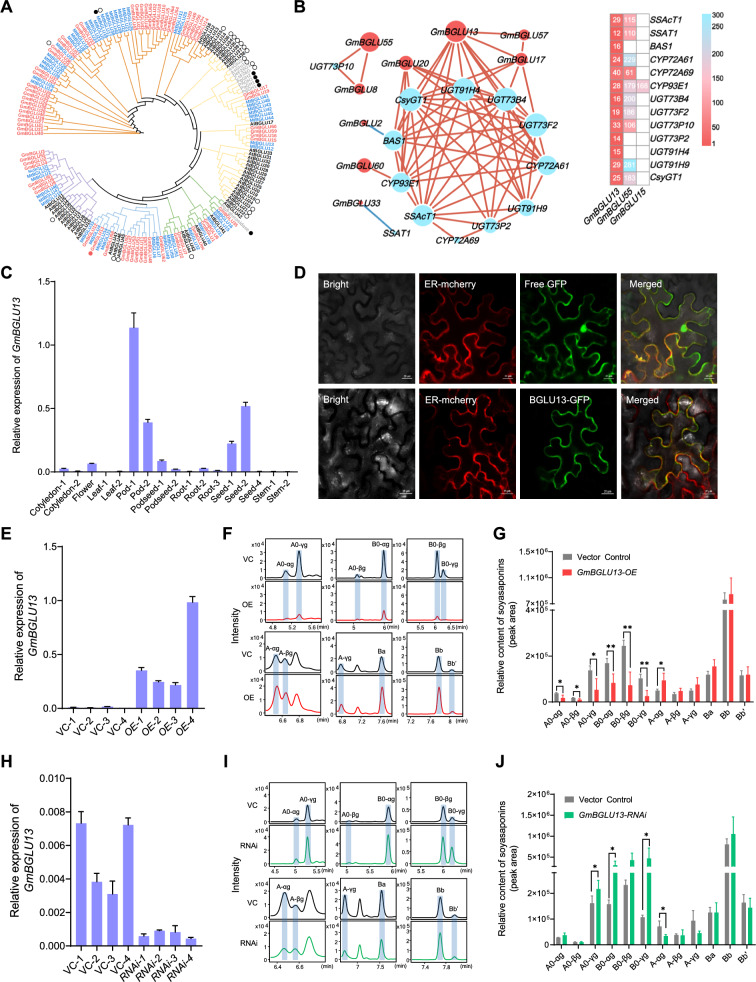
Determination of the candidate BGLU genes involved in soyasaponin homeostasis in soybean plants. **A** Phylogenetic analysis of BGLU proteins from soybean (red, 66 members), Arabidopsis (black, 47 members) and *Medicago truncatula* (blue, 51 members). Nine functional known BGLU proteins involved triterpene biosynthesis from other species also included in this analysis. The different-colored branches indicate different groups of plant BGLU proteins. The black solid circles and hollow circles represent triterpenoid BGLUs and other functional known BGLUs, respectively. The red solid circles represent the newly characterized GmBGLU13 in this study. **B** Determination of SSBG candidate genes by gene co-expression analysis. A total 66 soybean BGLU genes were used in both analyses. The transcriptome data set (left, representing 17 soybean tissues) was downloaded from Phytozome 13 (https://phytozome-next.jgi.doe.gov). Only Pearson's correlation coefficients r > 0.8 were retained and are shown as lines. The dot size reflects the number of connecting edges. Blue dots represent known soyasaponin biosynthesis genes, and red dots represent soybean BGLU genes. The right table was generated using the gene co-expression data set from ATTED-II 11.1 (https://atted.jp); the numbers represent the rank of correlation between the indicated pairs of known soyasaponin biosynthesis genes, with the highest r value taking a rank of 1; white squares have a rank ≥ 300. **C** Tissue specificity of expression for *GmBGLU13*. Values are means ± SD from three independent experiments. **D** Subcellular localization of GmBGLU13 protein. The ER-mcherry and BGLU13-GFP protein expressed in tobacco leaf cells. The endoplasmic reticulum (ER) was revealed by mCherry marker protein (the first 29 amino acids of At1g21270). This experiment was repeated twice with similar results. Scale bars = 20 μm. **E** Relative expression of *GmBGLU13* in *BGLU13-OE* transgenic and control (empty vector) hairy roots. **F** Extracted ion chromatograms of A0- and B0-soyasaponins and their corresponding hydrolysis products in control and *BGLU13-OE* transgenic hairy roots. The accurate masses used for extracting ions are provided in Table S4. **G** Relative quantification of soyasaponins in control and *BGLU13-OE* transgenic hairy roots. **H** Relative expression of *GmBGLU13* in *BGLU13-RNAi* transgenic and control (empty vector) hairy roots. **I** Extracted ion chromatograms of A0- and B0-soyasaponins and their corresponding hydrolysis products in control and *BGLU13-RNAi* transgenic hairy roots. The accurate masses used for extracting ions are provided in Supplemental Table S4. **J** Relative quantification of soyasaponins in control and *BGLU13-RNAi* transgenic hairy roots. Data are presented as mean ± SD of four independent experiments. Asterisks indicate significant differences: *P < 0.05 and **P < 0.01 (two-tailed Student's *t-*test)

To investigate whether BGLUs are involved in the homeostasis of soybean saponins, we first identified a total of 66 BGLU-encoding genes in the soybean genome using 47 BGLUs from *Arabidopsis thaliana* as templates (Fig. 2A and Table S1) (Xu et al. 2004). We further employed co-expression analysis strategy to determine the *BGLU* candidate genes involved soyasaponin homeostasis in *Glycine max*. Thirteen functional elucidated soyasaponin biosynthesis pathway genes were used as bait genes to perform the analysis, including one oxidosqualene cyclase (*BAS1*), three cytochrome P450 genes (*CYP72A61*, *CYP72A69*, and *CYP93E1*), eight glycosyltransferase genes (*CSyGT1*, *SSAT1*, *UGT73B4*, *UGT73F2*, *UGT73P2*, *UGT73P10*, *UGT91H4*, *UGT91H9*), and one soyasaponin acetyltransferase (*SSAcT1*). In total, three *BLGU* genes (*BLGU13*, *BLGU17*, and *BLGU20*) were found to be correlated with at least two known soyasaponin biosynthesis genes based on transcriptome data from Phytozome (Fig. 2B and Table S2) (Goodstein et al. 2012). Among them, *GmBLGU13* (*Glyma.07G258700*) exhibited positive correlations with nine soyasaponin genes (Pearson's correlation coefficients r > 0.8). Moreover, at the genome-wide level, *GmBLGU13* was the only *BLGU* gene ranked among the top 50 genes associated with known soyasaponin biosynthesis genes when using data from the ATTED-II database (Table S3) (Obayashi et al. 2022). Reverse transcription quantitative polymerase chain reaction (RT-qPCR) analysis confirmed that *GmBGLU13* exhibits higher expression levels in pods and seeds compared to other tissues, aligning with the previously reported expression pattern of the *GmSSAcT1* gene (Fig. 2C) (Yuan et al. 2023). Protein localization predictions using WoLF PSORT (https://wolfpsort.hgc.jp/) and TargetP-2.0 (https://services.healthtech.dtu.dk/services/TargetP-2.0/) indicated that the GmBGLU13 protein (525 amino acids peptide) is localized in the endoplasmic reticulum (ER) and contains an N-terminal signal peptide of 24 amino acids. This ER localization was further validated through confocal microscopy assays in a tobacco leaf system (Fig. 2D).

To investigate the *in planta* functions of *GmBGLU13*, we utilized soybean hairy roots derived from the KF‐1 cultivar. The gene was overexpressed using the CaMV 35S promoter and knocked down through an RNA interference (RNAi) approach. RT‐qPCR analysis confirmed that the relative expression of *GmBGLU13* were significantly upregulated in *GmBGLU13‐OE* (overexpression) hairy roots and significantly downregulated in *GmBGLU13‐RNAi* hairy roots (Fig. 2E and H). We employed liquid chromatography coupled with quadrupole time‐of‐flight tandem mass spectrometry (LC-qTOF-MS/MS, LC–MS thereafter) to profile and identify soyasaponins in the transformed hairy roots. A total of 22 soyasaponins were identified by extracting the exact masses of quasi-molecular ions and analyzing their characteristic fragment ions. Notably, B0-αg (Ba-22-Arabinose), B0-βg (Bb-22-Arabinose), and B0-γg (Bb’-22-Arabinose) were reported for the first time in soybean, with their chemical structures and comprehensive MS/MS data provided in Fig. S1. LC–MS analysis showed that A0-series soyasaponins (A0-αg, A0-βg, A0-γg) and B0-series soyasaponins (B0-αg, B0-βg, B0-γg) were significantly reduced in *GmBGLU13‐OE* hairy roots compared to the control KF‐1 hairy roots (empty vector) (Fig. 2F and G). Conversely, the relative contents of A0-γg, B0-αg, and B0-γg soyasaponins were significantly higher in *GmBGLU13‐RNAi* hairy roots compared to the control (Fig. 2I and J). Notably, no significant differences were observed in other types of soyasaponins (Fig. S2) and isoflavones (Fig. S3) between the control and *GmBGLU13* transgenic hairy roots.

These findings suggest that the GmBGLU13 encoded by *Glyma.07G258700* hydrolyzes the A0-series and B0-series soyasaponins. Based on these results, we redesignated GmBGLU13 as GmSSBG1 (SoyaSaponin β-Glucosidase1).

### Biochemical characterization of recombinant GmSSBG1

**Fig. 3:**
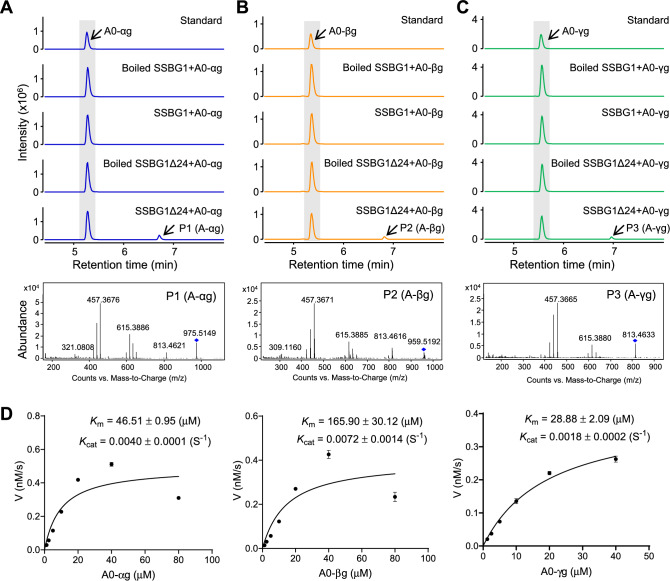
Biochemical characterization of GmSSBG1 in vitro. **A** LC–MS analysis of the products of reactions with recombinant SSBG1Δ24 using A0-αg as substrate. The mass spectrum of product was showed below. **B** LC–MS analysis of the products of reactions with recombinant SSBG1Δ24 using A0-βg as substrate. **C** LC–MS analysis of the products of reactions with recombinant SSBG1Δ24 using A0-γg as substrate. **D** Kinetic analysis of SSBG1Δ24 toward A0-αg (left), A0-βg (middle), and A0-γg (right). The reaction with a different type A0 soyasaponin for each data point was measured after 240 min of incubation. The apparent *K*_m_ and *K*_cat_ values for each tested soyasaponin are presented as the mean ± SD (*n* = 3)

To further investigate the function of GmSSBG1 in soyasaponin hydrolysis, we performed enzymatic activity assays using recombinant GmSSBG1 purified from *E. coli*. The full-length coding sequence (SSBG1) and a signal peptide-truncated version of GmSSBG1 (SSBG1Δ24) were cloned into the vector pMAL-c2x and expressed in *E. coli* BL21 (DE3) for in vitro protein production. Recombinant maltose-binding protein (MBP)-tagged SSBG1Δ24 was purified using amylose resin columns and confirmed by SDS-PAGE (Fig. S4). Since soyasaponin A0-series compounds are not commercially available, we prepared them in-house using a two-step procedure described in the Materials and Methods section and Fig. S5. The hydrolytic activity of recombinant SSBG1Δ24 towards A0-αg, A0-βg, and A0-γg was then assessed. A new peak (P1) was observed when A0-αg was incubated with recombinant SSBG1Δ24 (Fig. 3A). Mass spectrometry analysis revealed that the protonated molecule at *m/z* 975 [M + H]^+^ and fragment ions at *m/z* 813 [(M + H) − Glc]^+^, *m/z* 615 [(M + H) − Glc − Gal − 2H_2_O]^+^, and *m/z* 457 [(M + H) − Glc − Gal − H_2_O − GlcUA]^+^ of P1 were identical to those of A-αg (Fig. 3A), as reported previously (Krishnamurthy et al. 2019). Thus, P1 was identified as A-αg. Similarly, the product peaks P2 and P3 were detected when A0-βg and A0-γg were incubated with SSBG1Δ24, and these were identified as A-βg and A-γg, respectively.

Interestingly, the full-length version of SSBG1 exhibited no hydrolytic activity towards the three A0 soyasaponins (Fig. 3A–C). These results suggest that the N-terminal signal peptide is critical for enzymatic activity in the heterologous expression system. This observation aligns with a previous study, which reported that the N-terminal signal peptide of zeaxanthin epoxidase significantly influenced its catalytic activity (Cataldo et al. 2020). For simplicity, we refer to the signal peptide-truncated version SSBG1Δ24 as SSBG1 in the subsequent enzymatic assays.

Substrate specificity assays showed that SSBG1 exhibited no hydrolytic activity towards other types of soyasaponins or isoflavonoids (Table 1). Phylogenetic analysis revealed that SSBG1 is closely related to Arabidopsis BGLU45-47 (Fig. 2A), which are involved in lignification (Chapelle et al. 2012). However, when we tested the hydrolytic activity of SSBG1 against three monolignol glucosides and three isoflavonoids, no activity was detected (Table 1).

**Table 1:** Substrate specificity of GmSSBG1. The highest activity of GmSSBG1 toward A0-αg was set as 100%. All values obtained from this study are average of three independent enzymatic assays. N.D. not detectable

Chemical classification	Substrate name	Relative activity (%)
Soyasaponin (type A)	A0-αg	100
	A0-βg	65
	A0-γg	53
	Null-acetyled Ab	N.D
	Null-acetyled Af	N.D
	Null-acetyled Ac	N.D
	Aa	N.D
	Ab	N.D
	Ac	N.D
	Af	N.D
Soyasaponin (type B)	Ba	N.D
	Bb	N.D
	Bb’	N.D
Soyasaponin (type E)	Bd	N.D
	Be	N.D
Lignins	Syringin	N.D
	Coniferin	N.D
	*p*-Coumaryl alcohol 4-*O*-glucoside	N.D
Isoflavonoids	Daidzin	N.D
	Glycitin	N.D
	Genistin	N.D

We analyzed the pH and temperature preferences of GmSSBG1 using A0-αg as the substrate. GmSSBG1 exhibited maximum activity at pH 5.5, and the enzymatic activity peaked at 42 °C but dropped sharply at higher temperatures (Fig. S6). Additionally, the addition of 2 mM Ca^2+^ or Zn^2+^ increased activity by ~ 20%, while 2 mM Fe^3+^ or Cu^2+^ inhibited activity by over 60%. A time-course assay showed that the product formation was nearly linear from 1 to 4 h, but the reaction rate gradually decreased and almost ceased after 12 h (Fig. S6). Under optimal conditions, we determined the kinetic parameters of GmSSBG1. The *K*_m_ values were 46.51 µM for A0-αg, 165.90 µM for A0-βg, and 28.88 µM for A0-γg, comparable to MtG1 (Fig. 3D) (Lacchini et al. 2023) However, the *K*_cat_ values were significantly lower: 0.0040 s^−1^ for A0-αg, 0.0072 s^−1^ for A0-βg, and 0.0018 s^−1^ for A0-γg, compared to 4.05 s^−1^ for MtG1 (Fig. 3D) (Lacchini et al. 2023). These results indicate that GmSSBG1 is a relatively slow BGLU.

### *GmSSBG1* contributes to soybean pod borer resistance

**Fig. 4:**
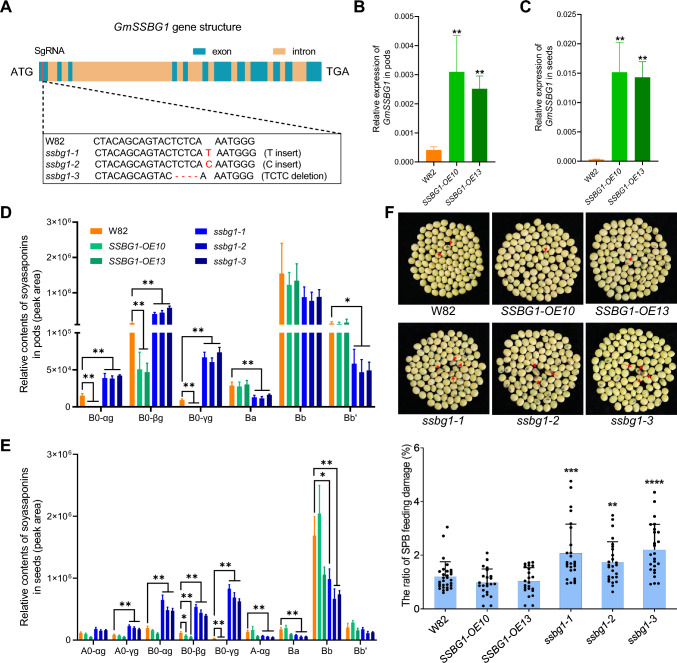
Characterization of *GmSSBG1* transgenic plants. **A** *ssbg1* mutants generated by CRISPR-Cas9-medidated gene editing technology. For representative sequence electrograms, see Supplemental Fig. S7. **B** Expression analysis of *GmSSBG1* in pods (R6 stage) of W82 (control) and two independent *SSBG1-*OE lines. **C** Expression analysis of *GmSSBG1* in developing seeds (R6 stage) of W82 and two independent *SSBG1-*OE lines. **D** Relative quantification of A0-, B0- A-, and B-series soyasaponins in pods (R6 stage) of W82 and Gm*SSBG1* transgenic lines. **E** Relative quantification of A0-, B0- A-, and B-series soyasaponins in developing seeds (R6 stage) of W82 and Gm*SSBG1* transgenic lines. Data are presented as mean ± SD of four independent experiments. Asterisks indicate significant differences: *P < 0.05 and **P < 0.01 (two-tailed Student's *t-*test). **F** Upper panel, representative images for SPB feeding damage on seeds in W82 and Gm*SSBG1* transgenic lines. Lower panel, evaluation of SPB resistance of W82 and Gm*SSBG1* transgenic lines. The SPB feeding damaged seeds were indicated by red arrows. Seeds from each plant were collected, and the ratio of SPB feeding damage was calculated in percentage (%). Asterisks indicate significant differences: **P < 0.01, ***P < 0.001, and ****P < 0.0001 (two-tailed Student's *t-*test)

To investigate the *bona fide* function of *GmSSBG1* in soybean, we generated three *GmSSBG1* knockout alleles using CRISPR-Cas9 technology in the Williams 82 background (Fig. S7). The *ssbg1-1* allele contained a single T base insertion at position 66 in the coding sequence (CDS), leading to the production of a prematurely terminated protein consisting of only 22 amino acids. Similarly, the *ssbg1-2* allele had a single C base insertion at the same position, resulting in a truncated protein of 45 amino acids. In contrast, the *ssbg1-3* allele featured a four-base deletion (TCTC) at positions 62–65 in the CDS, producing a prematurely terminated protein with 54 amino acids (Fig. 4A). To rule out the possibility of off‐target effects, we determined the top five predicted potential off‐target genes (*Glyma.18g199700*, *Glyma.20g202900*, *Glyma.01g242000*, *Glyma.11g002200*, and *Glyma.13g197100*) in all three knockout alleles. No BGLU homolog gene was included in the experiment based on DNA alignment, and no unexpected mutations were detected by sequencing (Fig. S8). Additionally, we generated two *GmSSBG1* overexpression alleles driven by the cauliflower mosaic virus (CaMV) 35S promoter, which verified by RT-PCR (Fig. 4B, C).

Given that *GmSSBG1* is primarily expressed in pods and seeds, we first quantified the relative soyasaponin contents in these tissues. Our analysis revealed that the levels of A0- and B0-series soyasaponins, which serve as substrates of GmSSBG1, were significantly higher in the knockout mutants and markedly lower in the overexpression alleles (Fig. 4D, E, Fig. S9 and S10). Like the results in transgenic hairy root, no significant differences were observed in other types of soyasaponins between the control and *SSBG1* transgenic plants (Fig. S11).

A recent study revealed that *GmSSBG1* was significantly enriched in response to SPB feeding in both SPB-resistant (JY93) and SPB-sensitive (K6) varieties (Chen et al. 2022). Combined with the tissue-specific expression pattern of *GmSSBG1*, this finding prompted us to investigate whether *GmSSBG1* contributes to SPB resistance. To test this hypothesis, *GmSSBG1*-overexpression (OE) and knockout (KO) lines were cultivated under natural field conditions. The extent of SPB feeding damage was assessed at soybean maturity. The results showed that the feeding damage ratio in the *ssbg1* mutant lines ranged from 1.7% to 2.2%, which was significantly higher than the 1.2% observed in W82 (control) plants (Fig. 4F). In contrast, *GmSSBG1*-OE lines displayed no significant differences, although slightly lower, in feeding damage compared to the W82. These findings demonstrate that *GmSSBG1* plays a critical role in soybean defense against SPB.

## Discussion

In summary, we report the identification of the first soyasaponin β-glucoside hydrolase, GmSSBG1 (GmBGLU13, encoded by *Glyma.07G258700*), from soybean plants. The biochemical function of GmSSBG1 in soybean, using A0- and B0-series soyasaponins as substrates, was elucidated through in vitro enzymatic assays, chemical analysis of hairy roots, and stable transgenic plants. However, we cannot exclude the possibility that other BGLUs, potentially outside the BGLU family (Minic 2008), may also contribute to soyasaponin homeostasis in soybean plants. This is suggested by the fact that key soyasaponins, including type-A, type-B, and DDMP-type, remained stable in GmSSBG1 transgenic plants, despite no other BGLUs being identified through gene co-expression analysis in this study.

Over the past decade, there has been significant progress in understanding the biosynthetic pathways of soyasaponins, particularly in relation to the enzymes and genes involved (Chung et al. 2020; Yuan et al. 2023). However, reports on the physiological roles of soyasaponins in soybean plants are still limited. This study demonstrates that *GmSSBG1* plays an important role in soybean resistance to the SPB. Based on chemical analysis, we propose that the direct products of GmSSBG1, A- and B-series soyasaponins with a single sugar moiety attached at the C3 position, contribute more significantly to this resistance than A0- and B0-series soyasaponins, which have two sugar moieties at both the C3 and C22 positions (Fig. 1). Similar structure–activity relationships have been reported in other plant species, such as torvoside A in turkey berry (*Solanum torvum*), avenacoside A and B in oat (*Avena sativa*), and 3-Glc-28-Glc-medicagenic acid in barrel medic (*Medicago truncatula*) (Osbourn 1996; Kim et al. 2000; Suthangkornkul et al. 2016; Lacchini et al. 2023). However, further studies are needed to identify the specific soyasaponins responsible for the direct deterrent or toxic effects on SPB. A key limitation of this study was the inability to obtain sufficient purified compounds. With advances in synthetic biology, including microbial and plant platforms, large-scale production and purification of rare soyasaponins is becoming increasingly feasible (Xu et al. 2020; Liu et al. 2023).

Currently, control of SPB primarily relies on chemical insecticides, which aim to directly kill the pest and reduce crop losses. However, excessive and prolonged use of insecticides not only leads to the development of pesticide resistance in the pest, diminishing control effectiveness, but also poses potential environmental and ecological risks (Hawkins et al. 2019). Genes that enhance plant insect resistance typically do so by producing insecticidal proteins, synthesizing specialized metabolites, and activating both local and systemic defense mechanisms (Falk et al. 2014; Heidel-Fischer and Vogel 2015; Kortbeek et al. 2019). Therefore, in-depth research on the role of soyasaponins and their involvement in plant defense mechanisms is essential. Such studies will not only uncover the molecular basis of SPB resistance in soybean plants, but also provide valuable insights and technological innovations for breeding insect-resistant crops. To our knowledge, a two-component chemical defense system has not been previously reported in soybean. Enhancing the efficiency of such a system to better protect against insect herbivory requires optimizing both the glycosylated substrates and the activating BGLU enzymes. Our results indicate that GmSSBG1 functions as a slow BGLU. Therefore, identifying superior haplotypes of GmSSBG1 with higher enzymatic activity from natural soybean populations for conventional resistance breeding (Fang et al. 2017; Chu et al. 2023), or employing a directed evolution strategy to improve the catalytic efficiency (*K*_*cat*_*/K*_*m*_) of GmSSBG1 presents a promising approach for strengthening soybean’s chemical defense against herbivores (Turner 2009; Ouyang et al. 2023).

## Materials and methods

### Chemicals

Soyasaponin standards Aa (CAS No.: 117230-33-8), Ba (CAS No.: 114590-20-4), and Bb (CAS No.: 51330-27-9) were purchased from Sigma‐Aldrich. Soyasaponin Ab (CAS No.: 118194-13-1) and Be (CAS: 117210-14-7) were obtained from Shanghai Tauto Biotech Co., Ltd (China). Soyasaponin Ac (CAS No.: 133882-74-3) and Af (CAS No.: 117230-32-7) were obtained from Chengdu Biopurify Phytochemicals Co., Ltd (China). Soyasaponin Bb’ (CAS No.: 55304-02-4) and Bd (CAS: 135272-91-2) Shanghai Acmec Biochemical Technology Co., Ltd (China). Lignin standard syringin (CAS No.: 118-34-3) was purchased from Shanghai Yuanye Bio-Technology Co., Ltd (China). Coniferin (CAS No.: 32811-40-8) was purchased from Shanghai Acmec Biochemical Technology Co., Ltd (China). *p*-Coumaryl alcohol 4-*O*-glucoside (CAS No.: 120442-73-1) was purchased from Sigma‐Aldrich. Isoflavones standards daidzin (CAS No.: 552-66-9), glycitein (CAS No.: 40246-10-4), and genistin (CAS No.: 529-59-9) were purchased from Sigma‐Aldrich. Commercially available BGLU (CAS No.: 9001-22-3, from almonds) was purchased from Sigma‐Aldrich.

The soyasaponin A0-series were prepared by a two-step manner as shown in Fig. S5. About 400 mM soyasaponin Ab or Ac or Af (final concentration) was deacetylated in alkaline solution (500 mM NaOH in 80% menthol aqueous solution) at room temperature overnight (Gu et al. 2002), then neutralized with an equivalent volume of formic acid solution. The mixture was evaporated and dissolved in 80% methanol aqueous solution. Subsequently, 40 µM (final concentration) deacetylated soyasaponin was added to 50 mM Na_2_HPO_4_-citric acid buffer (containing 5 mg/mL almonds BGLU, pH 5.4) and placed at 37 °C, overnight. The reaction was stopped by adding equivalent volume of methanol. The supernatant was collected, evaporated and dissolved in 80% methanol aqueous solution. Both steps are specific and the conversion rate from soyasaponin Ab (Ac or Af) to corresponding A0-αg (A0-βg or A0-γg) was almost 100% (Fig. S5).

### Plant materials

Soybean cultivar ‘KF-1’ (Aa type) was used to generate the transgenic hairy roots as described previously (Yuan et al. 2023). After infection, soybean plants were firstly grown in vermiculite for 2 weeks, and then transferred to tap water for 2 additional weeks after removing the main roots. Hairy roots were harvested and frozen immediately with liquid nitrogen and stored at − 80 °C before further analysis.

The *ssbg1* mutant plants were generated in the Williams 82 background (W82) using CRISPR-Cas9 technology. The gene editing process was carried out by WIMI Biotechnology Co., Ltd (Changzhou, China).

### Identification of GmBGLU genes of GH1 family in the *G. max* genome

The genome sequences and deduced protein sequences of *G. max* were downloaded from the SoyBase database (https://www.soybase.org/resources/). To identify *G. max* BGLU candidates of GH1 family (BGLU was referred as GH1 family hereinafter), two steps were carried out. First, Hidden Markov Model (HMM) profiles of Glyco_hydro_1 (PF00232) were downloaded from Pfam protein family database (https://pfam.xfam.org/) and used as the queries (P < 1e^−5^) to search the *G. max* protein sequence data. Second, BGLU protein sequences of *Arabidopsis* were downloaded from the TAIL database (https://www.arabidopsis.org/) and utilized as the query files against the *G. max* protein sequences with e-value ≤ 1e^−5^. Finally, 66 candidate *GmBGLU* genes were obtained and assigned based on their locations on chromosome (Table S1).

### Chemical analysis

Soybean samples were ground into powder in liquid nitrogen and freeze‐dried with lyophilizer. For hairy roots and hypocotyls, 6 mg powder was precisely weighed. 10 mg, 20 mg, and 30 mg powder were precisely weighed for seeds, pods, and leaves, respectively. The chemical analysis was performed on Agilent 1290 HPLC coupled to a 6550 qTOF-MS and absolutely identical to our previous investigation (Yuan et al. 2023). Detailed information about the chemicals analyzed by LC–MS is provided in Table S4.

### Gene cloning, expression and recombinant protein purification

The full‐length coding sequence of *GmSSBG1* was amplified using soybean pod cDNA as template. The full-length coding sequence of *SSBG1* and N-terminal 24 amino acids truncated version (*SSBG1Δ24*) were cloned into vector pMAL-c2x and validated by sequencing. Then the constructs were transformed into *E. coli* BL21(DE3) to express proteins. Aliquot of 5 mL *E. coli* cells harboring the recombinant plasmids was added into 500 mL liquid Luria–Bertani (LB) medium containing 100 µg/mL ampicillin. The cells were shaken at 37 °C and 230 rpm until optical density (OD) at 600 nm reached 0.6–0.8. A final concentration of 0.3 mM isopropyl 1-thio-β-D-galactoside (IPTG) was added to induce the expression of recombinant proteins, followed by incubation at 120 rpm and 16 °C for 24 h. Then cells were harvested and resuspended in buffer A (20 mM Tris–HCl, 200 mM NaCl, 10 mM β-Mercaptoethanol, pH 7.4), followed by sonication on ice for 15 min. After centrifuging at 12, 000 × *g* for 15 min at 4 °C, the supernatants were collected and purified by amylose resin column. The expression of proteins was confirmed by SDS-PAGE. Detailed primer information see Table S4.

### Enzymatic assays

The hydrolysis assays were conducted in a 50 µL reaction mixture containing buffer B (50 mM Na_2_HPO_4_-citric acid buffer, pH 5.5), 0.8 µg purified recombinant protein, and 5 µM substrates (A0-αg, A0-βg or A0-γg). The reaction mixture was incubated at 37 °C for 4 h and stopped by adding 50 µL methanol. Then the reaction mixture was centrifuged at 15,000 × *g* for 10 min before LC–MS analysis.

To determine the optimal pH and temperature, a series of buffer B (pH ranged from 3.0 to 8.0) and reaction temperature (between 25 °C and 60 °C) were tested. To determine whether the hydrolysis activity was affected by ions, 2 mM different ions were added. The reaction mixtures were incubated for 1–24 h to clarify the change of product over time. To calculate kinetic parameters, the enzyme assays were performed with different concentrations (1.25, 2.5, 5, 10, 20, 40, and 80 μM) of substrates (A0-αg, A0-βg or A0-γg) under the optimal pH and temperature. The reactions were initiated by adding substrates and incubated for 4 h. A0-αg, A0-βg, and A0-γg were used as standards for quantitative analysis of A-αg, A-βg, and A-γg, respectively. All the enzyme assays were in three replicates. The *K*_m_ and *K*_cat_ values were calculated using the Hyper 32 software.

### Subcellular localization of GmBGLU13

The subcellular localization of GmBGLU13-GFP was carried out following a previous published protocol (He et al. 2019).

### Molecular phylogenetic analysis

The amino acid sequences of 47 AtBGLUs, 51 MtBGLUs, 66 newly identified GmBGLUs and nine previously reported saponin hydrolase genes were used for phylogenetic analysis. All of these sequences were firstly aligned by using Muscle with the default parameters. Subsequently, an unrooted neighbor-joining phylogenetic tree was constructed based on the maximum-likelihood method (with 1,000 bootstrap replicates) using IQ-TREE version 2.0 (Minh et al. 2020). All plant BGLU proteins used in this analysis are listed in Table S1.

### Determination of SPB damage and statistical analysis

This experiment was conducted at the experimental fields of Northeast Agricultural University, which have been used extensively for SPB feeding trials and maintain a stable SPB population (Fang et al. 2024), ensuring natural feeding behavior. The study site is located at the university’s experimental station in Harbin, Heilongjiang Province (45°74′N, 126°72′E). The growing season extends from May to November, with an accumulated active temperature of 2,700 °C and a relative humidity of 67%. The soil at this location is classified as black soil. The average SPB density is approximately 5 individuals/m^2^. The experimental materials comprised two independent transgenic soybean lines (*SSHG1-OE10* and *SSBG1-OE13*), three mutant lines (*ssbg1-1*, *ssbg1-2*, and *ssbg1-3*), and the W82. The field experiment followed a randomized complete block design (RCBD), with three rows of each line planted per plot and three replicates. Each row contained 20 seeds, measured 1.4 m in length, and was spaced 0.6 m apart, with a 0.07 m gap between plants. Standard agronomic practices were implemented throughout the growing season to ensure the reliability of the experimental data. The soybean pod borer naturally feeds on soybeans in the field. At maturity, seeds from individual plants were harvested, and the number of seeds damaged by SPB was recorded to calculate the feeding damage percentage.

Statistical analysis was performed using a two-tailed Student’s *t*-test to evaluate differences in feeding rates among transgenic and W82 lines, with statistical significance set at *P* < 0.05. Data visualization was also performed using GraphPad Prism 8.0, and the results were expressed as mean ± standard deviation.

## Supplementary Information

Below is the link to the electronic supplementary material.

Supplementary file1 (DOCX 2237 KB)

Supplementary file2 (XLSX 25 KB)

Supplementary file3 (XLSX 30 KB)

Supplementary file4 (XLSX 218 KB)

Supplementary file5 (XLSX 15 KB)

Supplementary file6 (XLSX 11 KB)

## Data Availability

All data and materials generated during this study are available from the corresponding authors upon request.
