# Correction: Soyasaponin β-glucosidase confers soybean resistance to pod borer (*Leguminivora glycinivorella*)

**DOI:** 10.1007/s42994-025-00219-2

**Published:** 2025-08-05

**Authors:** Chengyong Feng, Xindan Xu, Jia Yuan, Mingyu Yang, Fanli Meng, Guodong Wang

**Affiliations:** 1 State Key Laboratory of Seed Innovation, Institute of Genetics and Developmental Biology, Chinese Academy of Sciences, Beijing, 100101 China; 2 State Key Laboratory of Black Soils Conservation and Utilization, Key Laboratory of Soybean Molecular Design Breeding, Northeast Institute of Geography and Agroecology, Chinese Academy of Sciences, Harbin, 150081 China; 3 College of Advanced Agricultural Sciences, University of Chinese Academy of Sciences, Beijing, 100039 China; 4 Present Address: Kunming Institute of Botany, Chinese Academy of Sciences, Kunming, 650201 China

**Correction: aBIOTECH** 10.1007/s42994-025-00214-7

The original article contained several errors caused in the production process of the article:

In this article the affiliation ‘State Key Laboratory of Seed Innovation, Institute of Genetics and Developmental Biology, Chinese Academy of Sciences, Beijing, 100101, China’ for Author Jia Yuan was missing.

Greek letters and other symbols were missing in Figs. 1 and 4 have now been included.

Fig. 1 with missing characters:

**Fig. 1:**
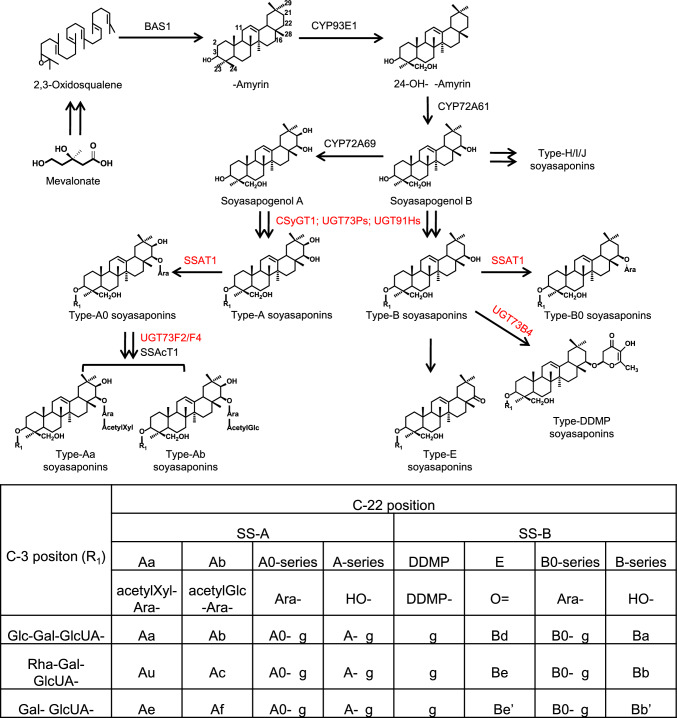
Proposed biosynthetic pathways of soyasaponins catalyzed by characterized enzymes in *Glycine max* L. The enzymes catalyzes the glycosylation reactions were highlighted in red. Double arrows represent multiple reactions. BAS1, β-amyrin synthase; CSyGT1, cellulose-synthase superfamily-derived glycosyltransferase1; SSAT1, soyasaponin arabinosyltransferase1; SSAcT1, soyasaponin acetyltransferase1

Correct Fig. 1 can be seen below:

**Fig. 1:**
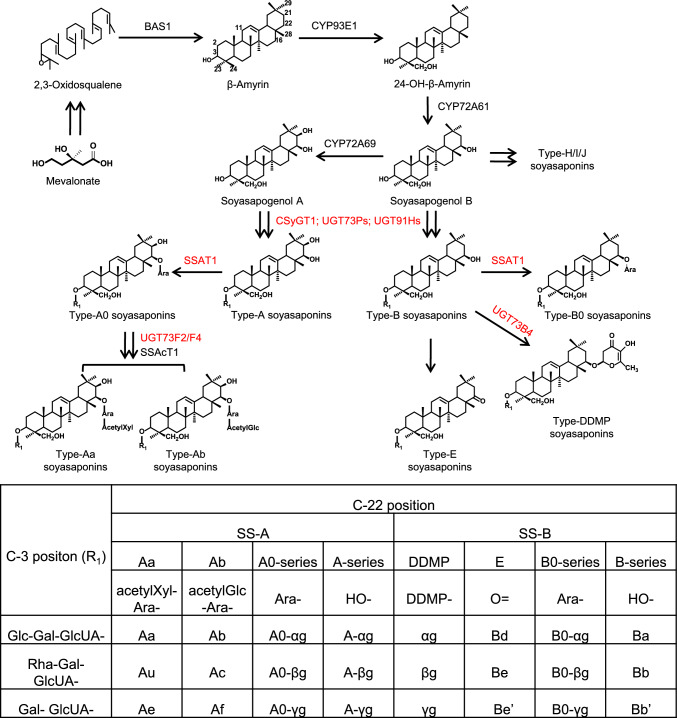
Proposed biosynthetic pathways of soyasaponins catalyzed by characterized enzymes in *Glycine max* L. The enzymes catalyzes the glycosylation reactions were highlighted in red. Double arrows represent multiple reactions. BAS1, β-amyrin synthase; CSyGT1, cellulose-synthase superfamily-derived glycosyltransferase1; SSAT1, soyasaponin arabinosyltransferase1; SSAcT1, soyasaponin acetyltransferase1

Figure 4 with missing characters:

**Fig. 4:**
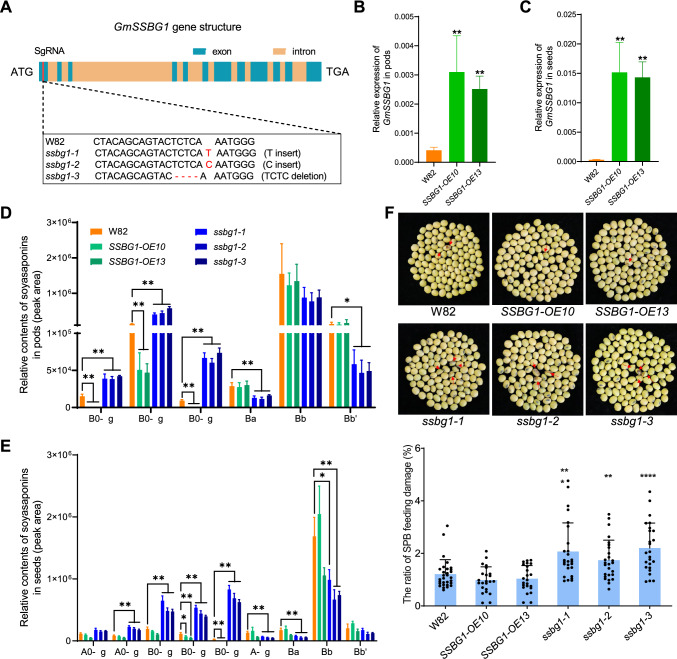
Characterization of *GmSSBG1* transgenic plants. **A** *ssbg1* mutants generated by CRISPR-Cas9-medidated gene editing technology. For representative sequence electrograms, see Supplemental Fig. S7. **B** Expression analysis of *GmSSBG1* in pods (R6 stage) of W82 (control) and two independent *SSBG1-*OE lines. **C** Expression analysis of *GmSSBG1* in developing seeds (R6 stage) of W82 and two independent *SSBG1-*OE lines. **D** Relative quantification of A0-, B0- A-, and B-series soyasaponins in pods (R6 stage) of W82 and Gm*SSBG1* transgenic lines. **E** Relative quantification of A0-, B0- A-, and B-series soyasaponins in developing seeds (R6 stage) of W82 and Gm*SSBG1* transgenic lines. Data are presented as mean ± SD of four independent experiments. Asterisks indicate significant differences: *P < 0.05 and **P < 0.01 (two-tailed Student's *t-*test). **F** Upper panel, representative images for SPB feeding damage on seeds in W82 and Gm*SSBG1* transgenic lines. Lower panel, evaluation of SPB resistance of W82 and Gm*SSBG1* transgenic lines. The SPB feeding damaged seeds were indicated by red arrows. Seeds from each plant were collected, and the ratio of SPB feeding damage was calculated in percentage (%). Asterisks indicate significant differences: **P < 0.01, ***P < 0.001, and ****P < 0.0001 (two-tailed Student's *t-*test)

Correct Fig. 4 can be seen below:

**Fig. 4:**
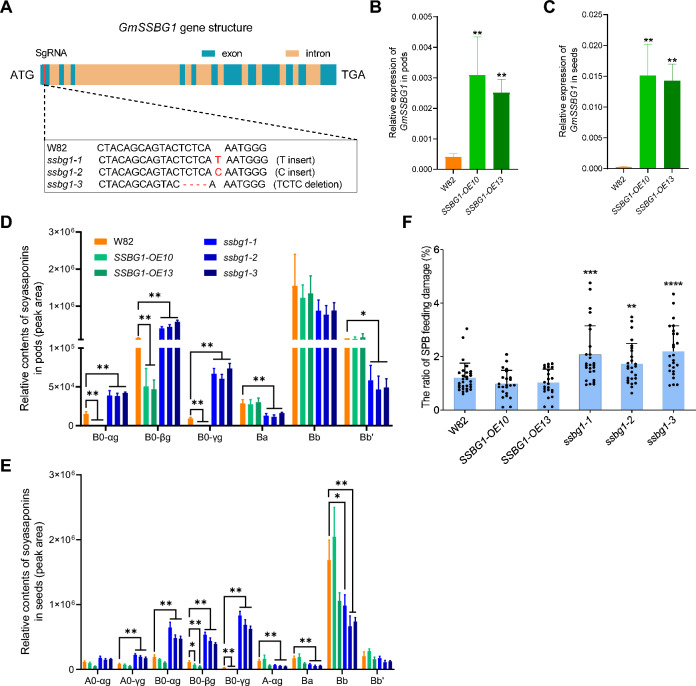
Characterization of *GmSSBG1* transgenic plants. **A** *ssbg1* mutants generated by CRISPR-Cas9-medidated gene editing technology. For representative sequence electrograms, see Supplemental Fig. S7. **B** Expression analysis of *GmSSBG1* in pods (R6 stage) of W82 (control) and two independent *SSBG1-*OE lines. **C** Expression analysis of *GmSSBG1* in developing seeds (R6 stage) of W82 and two independent *SSBG1-*OE lines. **D** Relative quantification of A0-, B0- A-, and B-series soyasaponins in pods (R6 stage) of W82 and Gm*SSBG1* transgenic lines. **E** Relative quantification of A0-, B0- A-, and B-series soyasaponins in developing seeds (R6 stage) of W82 and Gm*SSBG1* transgenic lines. Data are presented as mean ± SD of four independent experiments. Asterisks indicate significant differences: *P < 0.05 and **P < 0.01 (two-tailed Student's *t-*test). **F** Upper panel, representative images for SPB feeding damage on seeds in W82 and Gm*SSBG1* transgenic lines. Lower panel, evaluation of SPB resistance of W82 and Gm*SSBG1* transgenic lines. The SPB feeding damaged seeds were indicated by red arrows. Seeds from each plant were collected, and the ratio of SPB feeding damage was calculated in percentage (%). Asterisks indicate significant differences: **P < 0.01, ***P < 0.001, and ****P < 0.0001 (two-tailed Student's *t-*test)

Furthermore, Supplemental Figures file was revised and Supplemental Table 2 was missing and has now been added to the Supplementary Information section of the article.

## Supplementary Information

Below is the link to the electronic supplementary material.

Supplementary file1 (DOCX 2236 KB)

Supplementary file2 (XLSX 29 KB)

