# Supplementary material for: Soyasaponin β-glucosidase confers soybean resistance to pod borer (Leguminivora glycinivorella)
Source: aBIOTECH. 2025 May 10;6(2):160–73. doi: 10.1007/s42994-025-00214-7 (PMC12238454; doi:10.1007/s42994-025-00214-7)
Supplement: Supplementary file 1 — Supplementary file1 (DOCX 2237 KB) [file 42994_2025_214_MOESM1_ESM.docx]

**Supplemental Data (Total 11 Figures)**

**Soyasaponin β-Glucosidase Confers Soybean Resistance to Pod Borer (*Leguminivora glycinivorella*)**

Chengyong Feng^1^, Xindan Xu^1,3^, Jia Yuan^1,4^, Mingyu Yang^2^, Fanli Meng^2*^, Guodong Wang^1,3*^

^1^ State Key Laboratory of Seed Innovation, Institute of Genetics and Developmental Biology, Chinese Academy of Sciences, Beijing 100101, China;

^2^ State Key Laboratory of Black Soils Conservation and Utilization, Key Laboratory of Soybean Molecular Design Breeding, Northeast Institute of Geography and Agroecology, Chinese Academy of Sciences, Harbin 150081, China;

^3^ College of Advanced Agricultural Sciences, University of Chinese Academy of Sciences, Beijing 100039, China

^4^ Current address: Kunming Institute of Botany, Chinese Academy of Sciences, Kunming 650201, China

^*^ To whom correspondence should be addressed: gdwang@genetics.ac.cn and mengfanli@neau.edu.cn

**
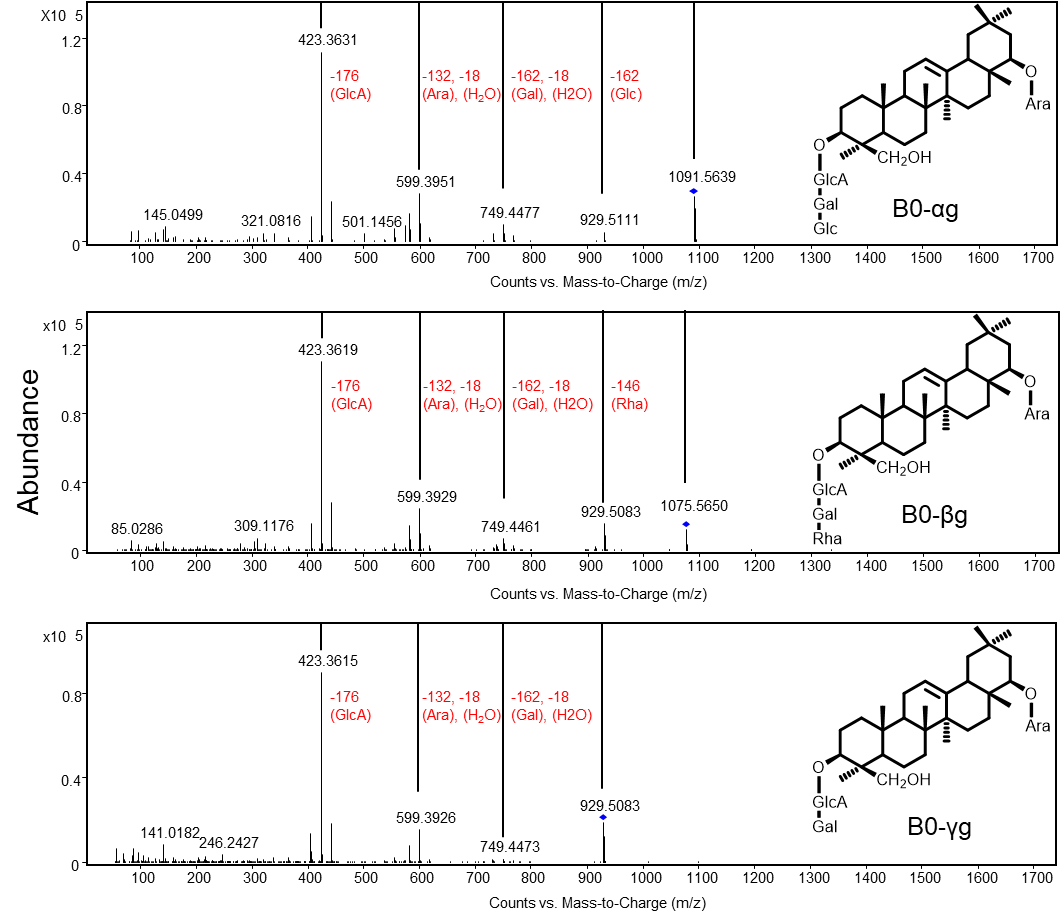
**

**Supplemental Figure 1.** Chemical structures and MS/MS data of three newly identified B0-soyasaponins discovered in this study. B0-αg, *m/z* 1091.5639 (upper panel); B0-βg, *m/z* 1075.5650 (middle panel); B0-γg, *m/z* 929.5083 (lower panel)

**
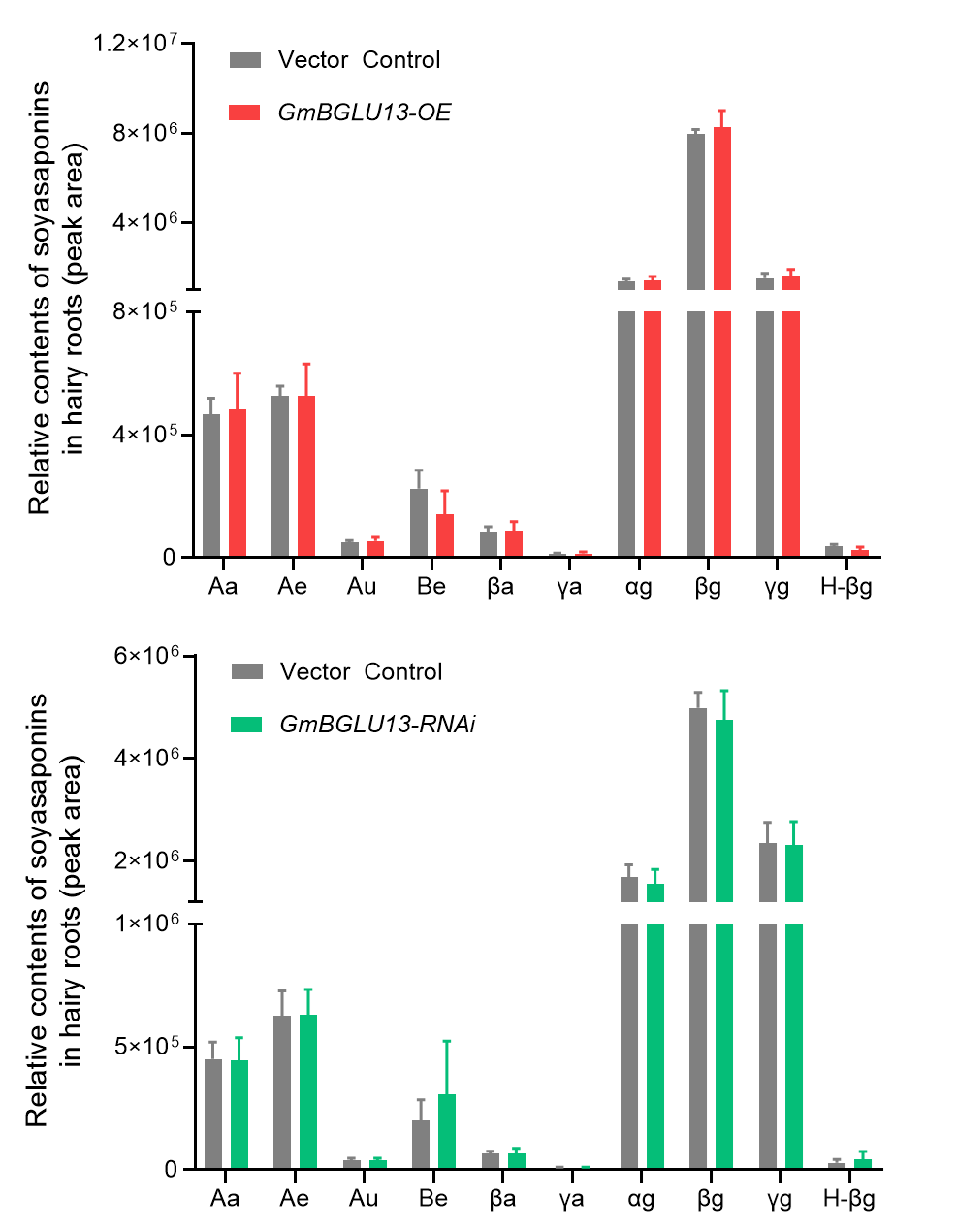
**

**Supplemental Figure 2.** Relative contents of other soyasaponins in *GmBGLU13*-overexpressing (OE) and knockdown (RNAi) hairy roots. The accurate masses used for ion extraction are listed in Table S4

**
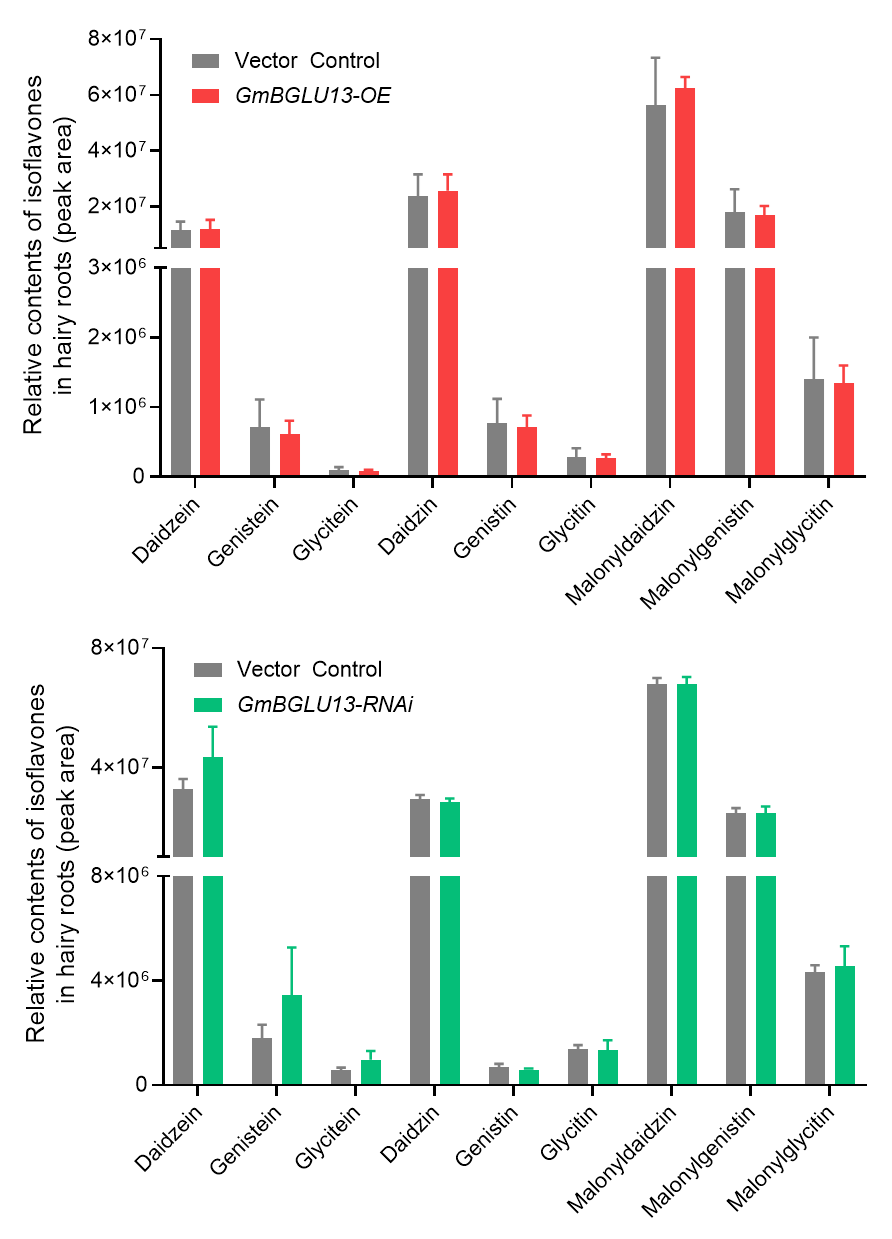
**

**Supplemental Figure 3.** Relative contents of other isoflavonoids in *GmBGLU13*-overexpressing (OE) and knockdown (RNAi) hairy roots. The accurate masses used for ion extraction are listed in Supplemental Table 4

**
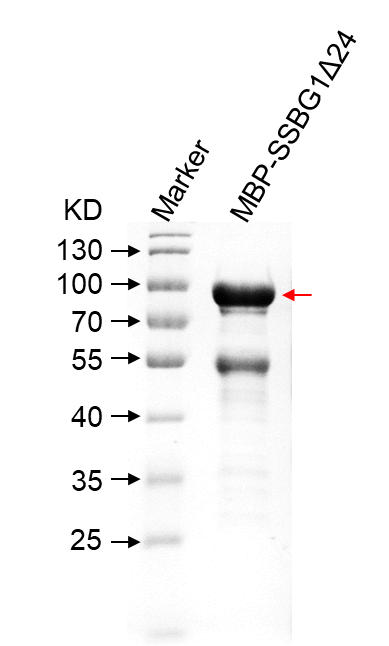
**

**Supplemental Figure 4.** SDS-PAGE analysis of purified recombinant GmSSBG1Δ24, marked with red arrow. The calculated molecular weight of recombinant MBP-GmSSBG1Δ24 is 101.03 KDa

**
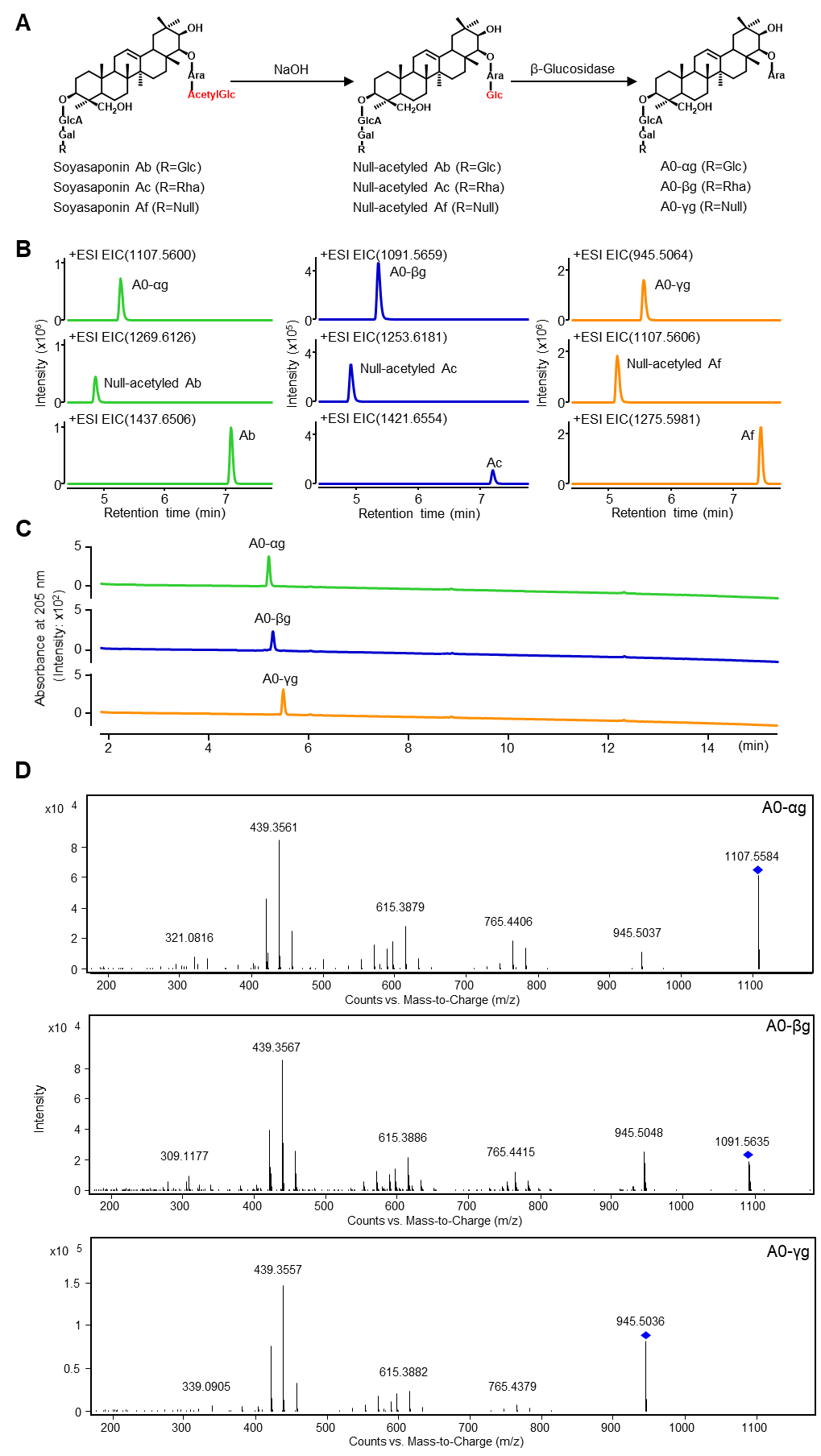
**

**Supplemental Figure 5.** Preparation of three types of A0-soyasaponins. A Schematic representation of the preparation process for three types of A0-soyasaponins. B Extracted ion chromatograms of soyasaponin standards and corresponding products. C HPLC chromatograms at 205 nm of the three prepared A0-soyasaponins. D MS^2^ spectra of three obtained A0-soyasaponins

**
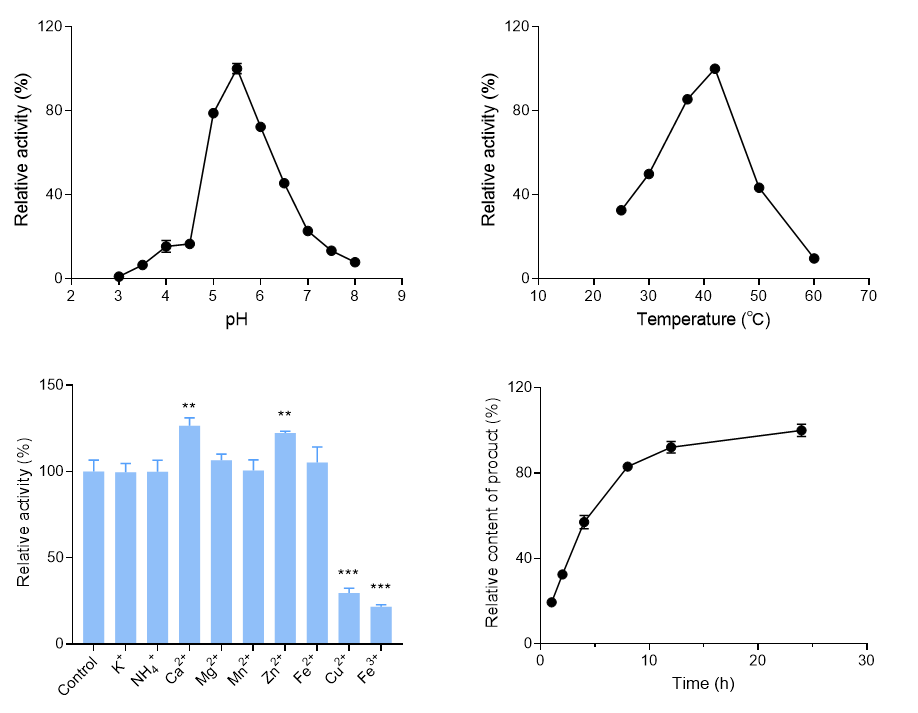
**

**Supplemental Figure 6.** Enzymatic characteristics of purified recombinant GmSSBG1Δ24, including optimal pH, optimal temperature, ideal ions, and reaction time

**
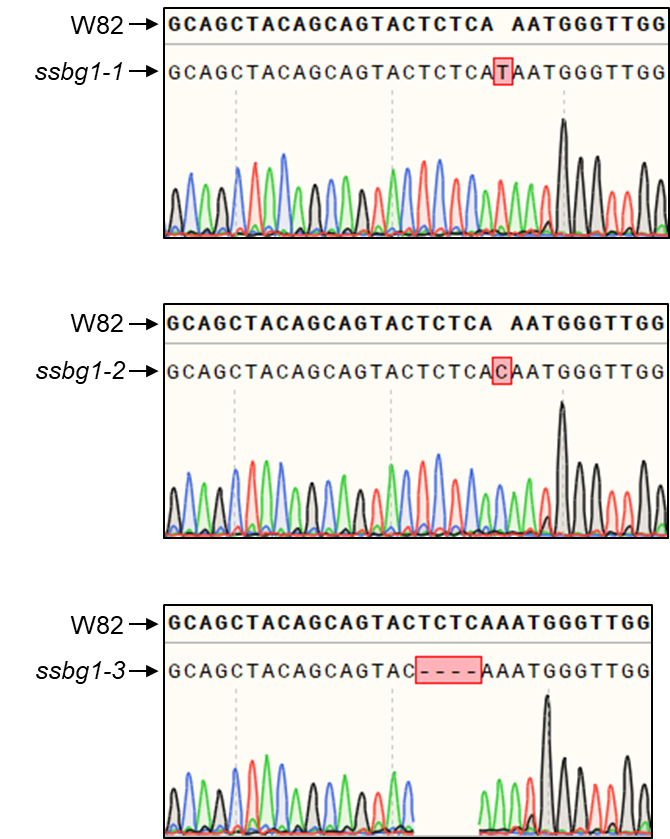
**

**Supplemental Figure 7.** Sequence alignment of the *GmSSBG1* gene in Williams 82 and three gene-edited alleles

**
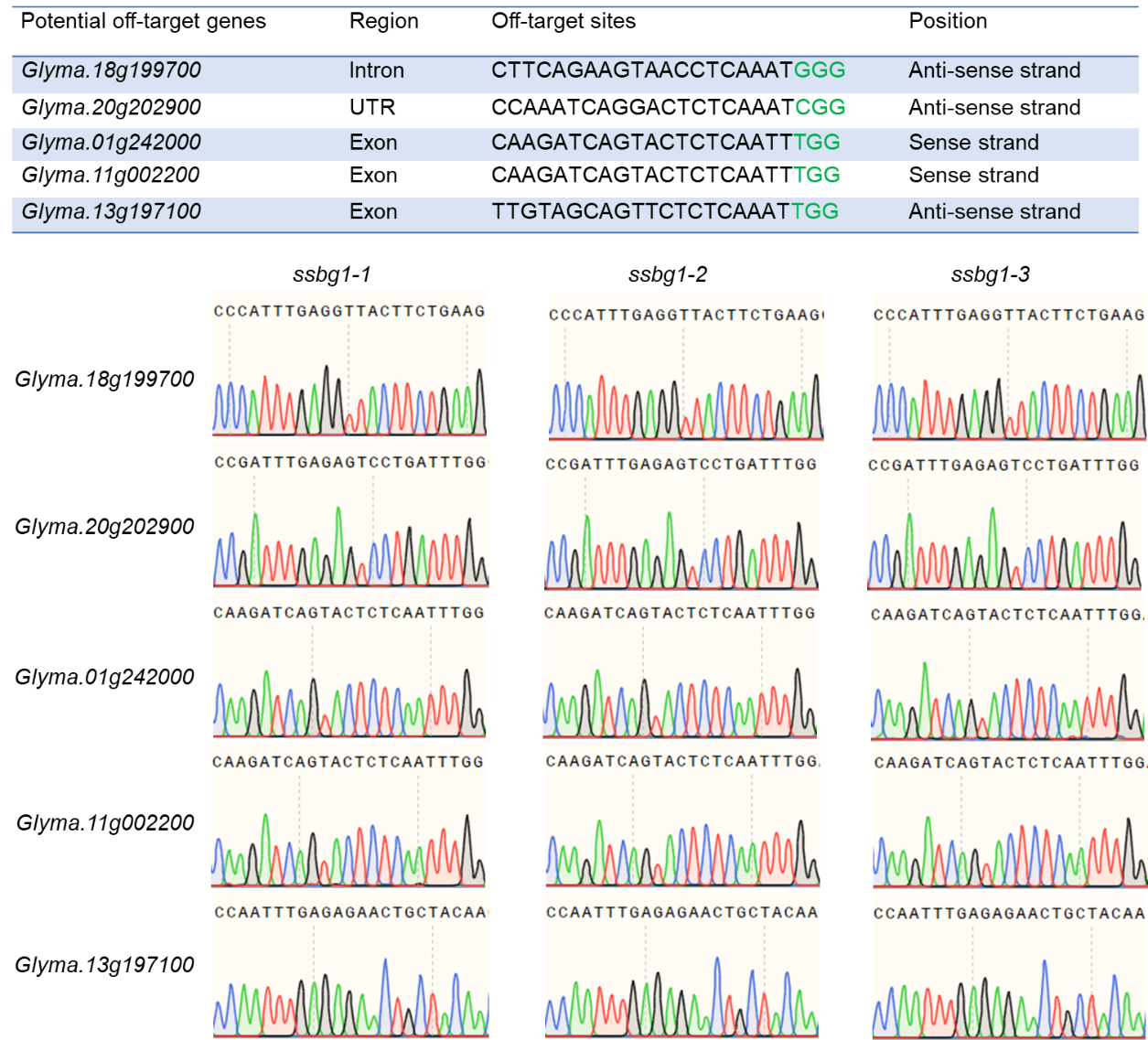
**

**Supplemental Figure 8.** Potential off-target effects using CRISPR-cas9 system through a single guide RNA approach. All five potential off-target genes were predicted using CRISPR-P 2.0 at the whole soybean genome level. PAM sequence (NGG) is highlighted in green

**
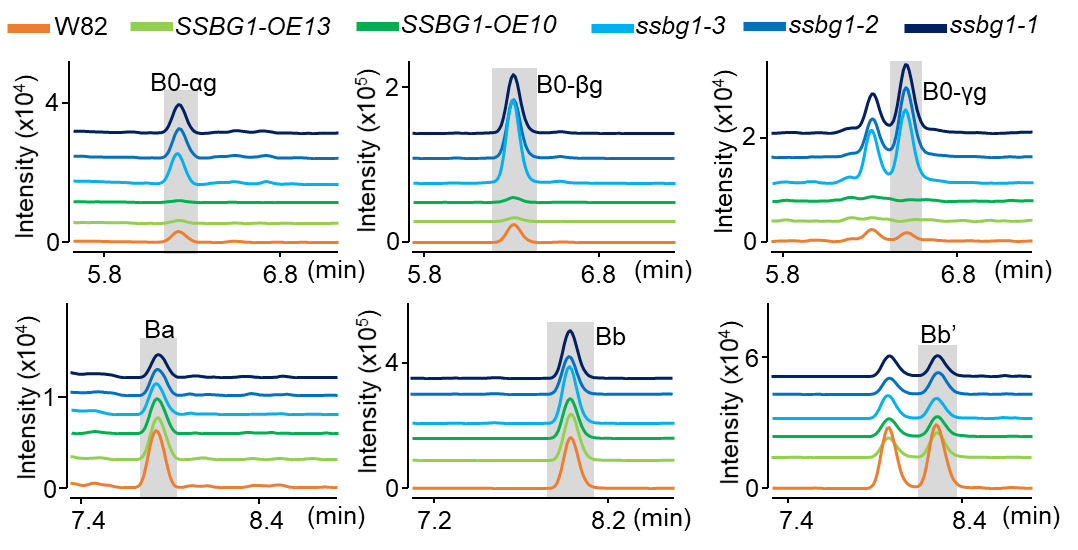
**

**Supplemental Figure 9.** LC-MS analysis of A0- and B0-series soyasaponins and their hydrolysis products in *SSBG1* transgenic soybean pods. The accurate masses used for ion extraction are provided in Table S4

**
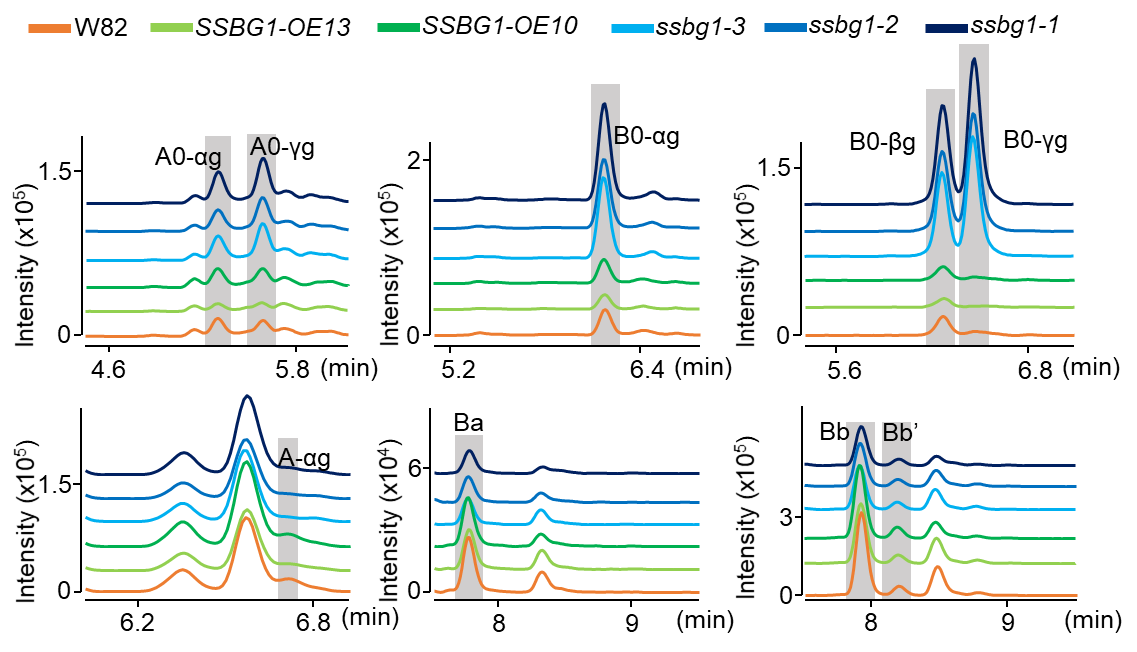
**

**Supplemental Figure 10.** LC-MS analysis of A0-and B0-series soyasaponins and their hydrolysis products in *SSBG1* transgenic soybean seeds. The accurate masses used for ion extraction are provided in Table S4.

**
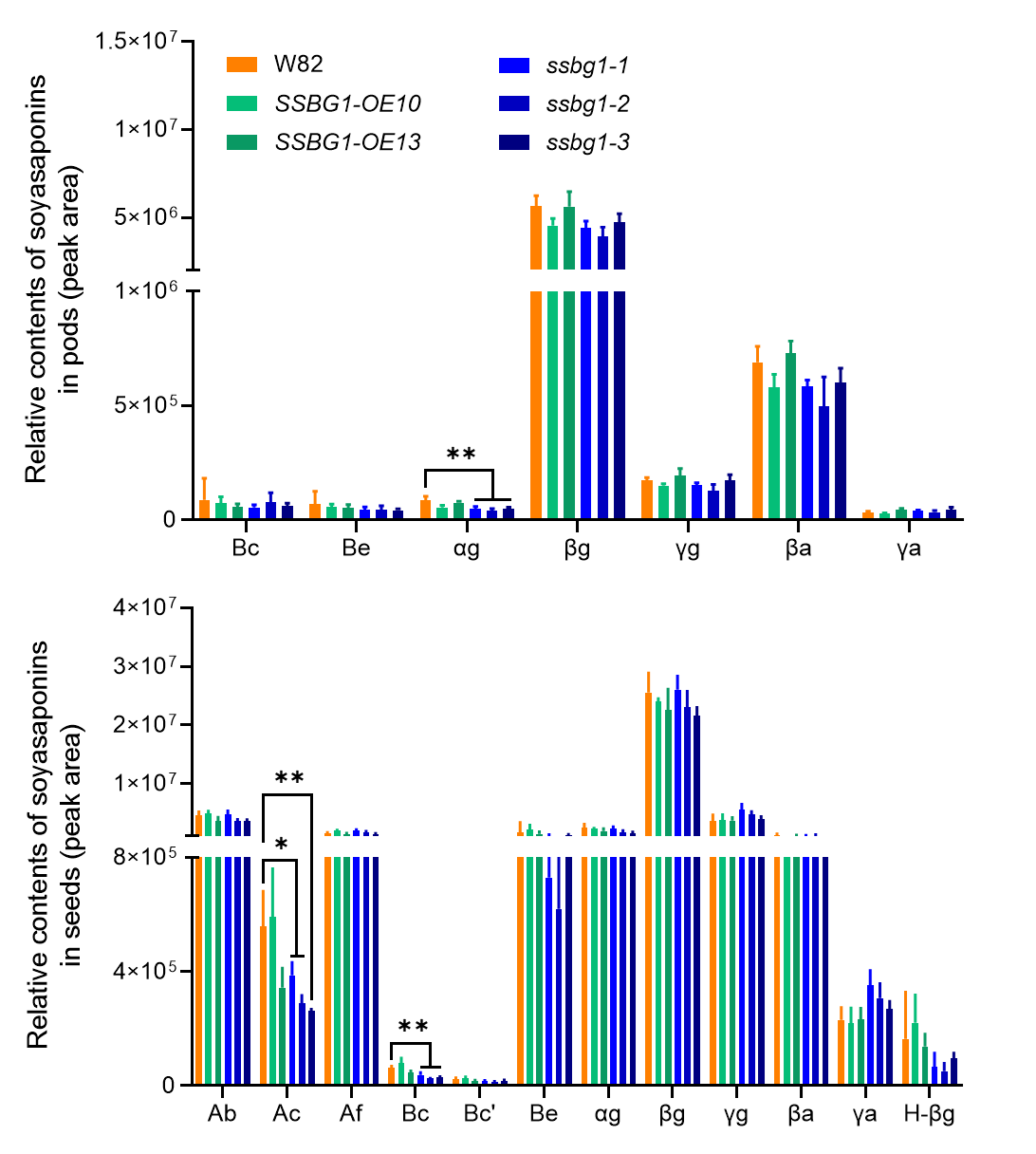
**

**Supplemental Figure 11.** Relative contents of other soyasaponins in SSBG1-overexpressing and knockout pods (upper panel) and seeds (lower panel). The accurate masses used for ion extraction are provided in Table S4.
